# Fluorescence amplified fragment length polymorphism compared to pulsed field gel electrophoresis for *Listeria monocytogenes* subtyping

**DOI:** 10.1186/1471-2180-13-14

**Published:** 2013-01-24

**Authors:** Sophie Roussel, Benjamin Félix, Kathie Grant, Trinh Tam Dao, Anne Brisabois, Corinne Amar

**Affiliations:** 1 ANSES, Maisons-Alfort Laboratory for Food Safety, Bacterial Characterization and Epidemiology Unit, 23, avenue du Général de Gaulle, MAISONS-ALFORT cedex, 94706, France; 2 Health Protection Agency, Gastrointestinal Bacteria Reference Unit, 61 Colindale Avenue, London, NW9 5EQ, UK

**Keywords:** fAFLP, PFGE, Molecular subtyping, *Listeria monocytogenes*, Discriminatory power

## Abstract

**Background:**

Listeriosis is a severe infection which mainly affects pregnant women, neonates and immuno-compromised adults. ANSES’s Laboratory for Food safety has been the European Union Reference Laboratory (EURL) for *L. monocytogenes* in the food chain since 2006. Pulsed Field Gel Electrophoresis (PFGE) is routinely used in the EURL for the surveillance of *L. monocytogenes* isolated from foods, animals and the environment. One of the main EURL activities is to evaluate alternative molecular subtyping methods to PFGE, and integrate their use within the National Reference Laboratories (NRL) network. Since 2008, the United Kingdom (UK)-NRL for *L. monocytogenes* at the Health Protection Agency (HPA), London, has used fluorescent Amplified Fragment Length Polymorphism (fAFLP) for the routine surveillance of *L. monocytogenes* isolated from human clinical cases, food and food processing environments in the UK. This study compares fAFLP with PFGE for subtyping *L. monocytogenes*.

**Results:**

A panel of 109 *L. monocytogenes* isolates from either human cases of listeriosis, foods, food processing environments and animals were used for the comparative evaluation. Among these, 2 strains were tested from duplicate culture by both methods. The panel also included field isolates, isolates associated with outbreaks or sporadic cases and reference strains. The two strains tested in duplicate displayed the same fAFLP and PFGE types. Strains known to be epidemiologically associated with one another were found to have unique PFGE and fAFLP types. FAFLP and PFGE divided the strains into 76 and 82 distinct profiles, or types, respectively. The discriminatory index calculated was 0.993 and 0.996 for fAFLP and PFGE, respectively.

**Conclusions:**

The discriminatory ability of fAFLP was similar to that of PFGE for the subtyping of *L. monocytogenes* isolates. As a less labour intensive technique fAFLP may be a better method to use than PFGE in investigating outbreaks of human listeriosis and tracking the source of contamination in food processing facilities in real time.

## Background

Listeriosis is a food borne disease caused by the bacterium *L. monocytogenes*. In otherwise healthy individuals, listeriosis is usually asymptomatic or may results in mild flu-like symptoms or gastrointestinal illness. However, infection with *L. monocytogenes* in pregnant women, neonates and immuno-compromised adults can result in a severe and life threatening invasive form of listeriosis. In Europe, after a decline in the number of cases during the 1990s, the incidence of listeriosis increased and has remained relatively high for the past ten years. This has led to listeriosis being considered one of the resurgent foodborne diseases in Europe [1,2]. This disease is rare but associated with a high fatality rate (20-30%) and currently remains an important public health concern.

Based on its genetic content, *L. monocytogenes* can be separated into 3 lineages I, II and III. Although 13 serotypes have been described, 98% of strains causing human infections and isolated from foods are of serotypes 4b, 1/2b (Lineage I), 1/2a, and 1/2c (lineages II) [3]. Molecular methods have been developed to assist in the characterization of *L. monocytogenes.* Doumith et al. (2004) [4] have described a multiplex PCR assay which cluster *L. monocytogenes* of lineages I and II into four serogroups: IVb (4b, 4d, 4e); IIa (1/2a, 3a), IIb (1/2b, 3b, 7) and IIc (1/2c, 3c).

Of several molecular methods currently available, macrorestriction analysis by PFGE is one of the most used methods for the subtyping of *L. monocytogenes*[5,6]. The combination of restriction endonucleases *Asc*I and *Apa*I, as advised by PulseNet USA, has shown excellent discrimination for *L. monocytogenes*[5] and the technique is shown to be reproducible. PFGE, using these two enzymes, is considered to be the international standard for subtyping [7].

AFLP is a method which combines the digestion of the entire bacterial genome by one or more restriction enzymes, with PCR being used to amplify and detect the digested fragments. Fluorescent AFLP is a variant using fluorescent PCR primers, enabling the amplified digested fragments to be detected and sized accurately by capillary electrophoresis. Various fAFLP assays have previously been developed for subtyping *L. monocytogenes* and other *Listeria* spp isolated from food, animals, food processing environment [8] and human cases [9,10]. These assays have been described as reproducible and high resolution genotyping techniques that require less time to perform and to analyze than PFGE. Recently, fAFLP with the enzyme pair *Hind*III/*Hha*I was applied to *L. monocytogenes* isolates from foods and the environment [11], using adaptors and primers previously designed [12] for typing *Campylobacter* isolates. This enzyme pair was found to be more suitable for *L. monocytogenes* than the *BamH*1/*EcoR*I pairs [13]. To our knowledge, these authors have compared, for the first time, fAFLP with PFGE combined with the two enzymes *Apa*I/*AscI* and demonstrated that the discrimination index (DI) of fAFLP was at least equal to PFGE. However, the strain panel only included field strains isolated from food and food processing environments and not human clinical isolates.

ANSES’s Laboratory for Food safety has been the EURL for *L. monocytogenes* in the food chain since 2006. *Apa*I/*AscI*-PFGE is routinely used at the EURL for the surveillance of food, animals and environmental isolates at the national and European level [14,15]. One of the main EURL activities is to develop or/and evaluate and keep up to date with new molecular subtyping methods and deploy them through the European NRL network. PFGE is widely acknowledged to be a time-consuming and labor-intensive method: the analyses are completed in 30 hours to three days from receipt of pure culture. It also requires highly skilled operators and does not offer commercially available standardized reagents. To consider a subtyping technique for *L. monocytogenes* as an alternative to PFGE, one of the first step is to test a panel of strains isolated not only from food and environment samples but also from human cases and to include outbreaks and reference strains [16].

Since 2008 the UK-NRL for *Listeria*has used fAFLP, with the enzyme pairs *Hind*III/*Hha*I, as the subtyping method for the routine surveillance of *L. monocytogenes* isolated from human clinical cases, food and food processing environments in the UK.

The objective of this study was to compare results obtained using fAFLP and PFGE, on a panel of *L. monocytogenes* isolates from human clinical cases, foods, food processing environments and animals. The panel included isolates known to be associated with outbreaks and sporadic cases of listeriosis, as well as reference strains, 3 of which were fully sequenced. The value of fAFLP for the routine subtyping of *L. monocytogenes*, in terms of its discriminatory ability and usefulness in detecting and investigating clusters of listeriosis cases for the NRLs, will be discussed.

## Methods

### Strains

This study included 109 isolates of *L. monocytogenes*: 47 from human cases of listeriosis, 56 from different food products and food processing environments, and 6 from animals. Strains in this study were selected to include those associated with listeriosis outbreaks as well as sporadic cases and were representative of the serogroups most often associated with human disease.

Forty nine isolates came from the UK-NRL: 35 were from UK clinical cases of listeriosis and 14 from foods and food processing environments isolated by UK-HPA Food Water and Microbiology Laboratories either as part of routine food sampling or in response to listeriosis investigations. One of the UK isolates from a clinical case of listeriosis was included in the study as duplicate culture (Table 1).

**Table 1:** **PFGE and fAFLP discriminatory ability using *Listeria monocytogenes *isolates of duplicate strains, associated with outbreaks or with sporadic cases**

**Isolate**	**Test Study (TS) group number**[17]	**Responsible for sporadic (S) or outbreak (OB). Duplicate culture (D)**	**Origin of isolate**	**Country of origin**	**Molecular serogroup**^**1**^	***PFGE***^***2***^***Apa*I*/Asc*I type**	**fAFLP**^***2***^***Hha*I/*Hind*III type**
10CEB565LM	n/a	OB 1	Human	England	IVb	326/136	IV4.3
10CEB567LM	n/a	OB 1	Food	England	IVb	326/136	IV4.3
10CEB550LM	n/a	OB 2	Human	England	IVb	178/6	I.8
10CEB552LM	n/a	OB 2	Food	England	IVb	178/6	I.8
10CEB553LM	n/a	OB 3	Human	England	IIa	149/109	III.10
10CEB554LM	n/a	OB 3	Food	England	IIa	149/109	III.10
10CEB559LM	n/a	OB 4	Human	England	IVb	309/142	UD4.1
10CEB560LM	n/a	OB 4	Food	England	IVb	309/142	UD4.1
10CEB542LM = 10CEB543LM^3^	n/a	D	Human	England	IIc	70/377	VIIc.8
TS32	02	S	Food	USA	IVb	180/50	I.67
TS72	02	S	Food	USA	IVb	180/50	I.67
TS56 = TS77^3^	03	S^4^ and D	Human	USA	IIa	120/191	VIIa.27
TS39	03	S	Food	USA	IIa	120/191	VIIa.27a
TS67	03	S^4^	Human	USA	IIa	120/191	VIIa.27a
TS17	05	S	Human	USA	IIb	93/140	IVb.21
TS61	05	S	Food	USA	IIb	93/140	IVb.21
TS31	15	OB 5	Human	France	IVb	24-Dec	V.21
TS69	15	OB 5	Human	France	IVb	24-Dec	V.21
TS21	16	OB 6	Food	Switzerland	IVb	19/15	V.3
TS55	16	OB 6	Human	Switzerland	IVb	19/15	V.3
TS02	22	S2^5^	Human	England	IIc	70/25	VIIc.1
TS08	22	S2^5^	Human	England	IIc	70/25	VIIc.1

^1^ Serogrouping performed by multiplex PCR [4]: results are from both the European Reference Laboratory (EURL) for *L. monocytogenes* and the UK National Reference laboratory (UK-NRL) for *Listeria.*

^2^ PFGE was performed by the EURL and fAFLP by UK-NRL.

^3^ Serogrouping and typing results were the same for each of the duplicate culture.

^4^ The 2 patients of TS group number 3 were 2 separate sporadic cases and not epidemiologically linked [18].

^5^ These 2 isolates are from the same patient who had 2 recurrent episodes of listeriosis [19].

n/a: not applicable.

Sixty one isolates came from the EURL: 35 were from foods and food processing environments collected from French food analysis laboratories in the context of monitoring, surveillance sampling or research projects and one was from an animal. Thirteen isolates labeled TS (“Test study”), 8 from human cases and 5 from foods, were from the WHO international multicenter *L. monocytogenes* subtyping study [17,20]. One TS strain from a human case of listeriosis was included in this study as duplicate culture (Table 1). Eleven isolates were reference strains including 8 CLIP strains and 3 fully sequenced strains (Table 2).

**Table 2:** **Origins and serogroups of 11 *L. monocytogenes *reference strains used in this study**

**Reference strains**	**EURL Strain number**	**Origin**	**Molecular serogroup**^**2**^
CLIP^1^ 74902	00EB248LM	Animal	IIa
CLIP 74903	00EB249LM	Animal	IIb
CLIP 74904	00EB250LM	Human	IIc
CLIP 74905	00EB251LM	Human	IIa
CLIP 74906	00EB252LM	Human	IIb
CLIP 74907	00EB253LM	Animal	IIb
CLIP 74910	00EB256LM	Environment	IVb
CLIP 74912	00EB258LM	Animal	IVb
EGDe	EGDe	Animal	IIa
(Accession number: AL591824)			
[21]			
F2365	F2365	Food	IVb
(Accession number: AE017262)			
[22]			
CLIP80459 [23]	CLIP80459	Human	IVb

^1^ CLIP: Pasteur Institute collection.

^2^ Serogrouping performed by multiplex PCR [4]: results are from both the European Reference Laboratory (EURL) for *L. monocytogenes* and the UK National Reference laboratory (UK-NRL) for *Listeria*.

### Molecular serogrouping

All the isolates were serogrouped by both laboratories using the multiplex PCR assay described by Doumith et al. (2004) [4] which clusters *L. monocytogenes* lineages I and II into four serogroups by amplification of four specific marker genes: *lmo*0737; *ORF*2110; *lmo*1118 and *ORF*2819.

### Fluorescent AFLP

FAFLP was performed by the UK-NRL using a modified version of the protocol previously described by Desai and colleagues for *Campylobacter*[12]. Briefly, *Listeria* genomic DNA (15–50 ng) was digested with 5U each of two restriction enzymes, *Hind*III and *Hha*I (New England Biolabs) in the presence of RNase A and bovine serum albumin. Digests were ligated to two sets of double-stranded adapters. These adapters served as targets for an FAM-labeled Hind-A and a non-labeled Hha-A selective primer (Eurogentec, Seraing) for fragment amplification by PCR. The modified protocol consisted of a single digestion/ligation rather than 3 individual steps as previously described [12]. Fluorescent PCR products (amplified digested fragments) were separated on an ABI 3730XL 96 capillary DNA Analyzer (Applied Biosystems) alongside a GeneScan™- 600 LIZ® Size standard. Chromatographs showing FAM-fluorescing fragments were saved as fsa files, and were exported, visualized and analyzed using PEAK SCANNER™ v1.0 (Applied Biosystems). PEAK SCANNER™ also recorded the fragment data in a binary format in Excel files which were exported into BioNumerics v6.1, visualized as virtual electrophoresis gels and analyzed. The patterns determining the fAFLP types were identified using in-house BioNumerics and PEAK SCANNER™ libraries. Two profiles were considered to be different fAFLP types if they had at least one peak difference.

### PFGE

All strains were characterized using the EURL protocol [14,15] using the two restriction enzymes *Apa*I and *Asc*I. The laboratory has been accredited by the French Accreditation Committee, COFRAC for this PFGE method as an internal method (Accreditation No. 1–2246, Section Laboratories, http://www.cofrac.fr). Fragments obtained from the digestion by each of the enzymes were separated by gel electrophoresis. Gels were stained with ethidium bromide and banding patterns visualized under UV light, using the Gel Doc Eq system and Quantity One software (Bio-Rad). DNA patterns generated were analyzed with BioNumerics software (V 6.1, Applied Maths, Kortrijk, Belgium). Algorithms available within the program were used to compare patterns. For each enzyme, dendrograms were produced, using the Dice coefficient and UPGMA, with a 1% tolerance limit and 1% optimization. The dendrogam settings were chosen according to the PulseNet Europe recommendation [24]. Profiles were analyzed according to the standard operating procedure (SOP) developed at the EURL [15]. PFGE profiles were classified as different if there was at least one band different between them. Each PFGE profile was arbitrarily assigned a number.

### Reproducibility of the subtyping methods

Two strains were included blindly as duplicates cultures (Table 1).

### Discriminatory power of the subtyping methods

The ability of the two subtyping methods to discriminate *L. monocytogenes* strains was assessed in two ways:

(1) Determining the ability of the typing methods to recognize strains that are epidemiologically linked (Table 1).

(2) Determining the ability of the typing methods to discriminate unrelated strains by calculating the Simpson’s index of diversity (ID) [25]. The ID was calculated from PFGE and FAFP results of 97 isolates comprising field strains (75 isolates), references strains (11 isolates), sporadic cases and one representative isolate from each of the outbreaks shown in Table 1 (11 isolates).

## Results

### Molecular serogrouping

Molecular serogrouping results from the 109 isolates were concordant between the two testing laboratories and were as follows: 46 IIa strains; 12 IIb strains; 10 IIc strains; 40 IVb strains. One isolate did amplify in the multiplex PCR assay and was subsequently serotyped by conventional sero-agglutination by EURL as 4a strain. The 11 reference strains (8 CLIP and 3 fully sequenced strains) were found to belong to the expected serogroup (Table 2).

In both laboratories, the same four serogroup IVb strains, displayed an unusual multiplex PCR profile to that usually observed with IVb strains, with an additional band due to the amplification of the *lmo*0737 gene fragment as previously described [26].

### Subtyping data

**Figure 1:**
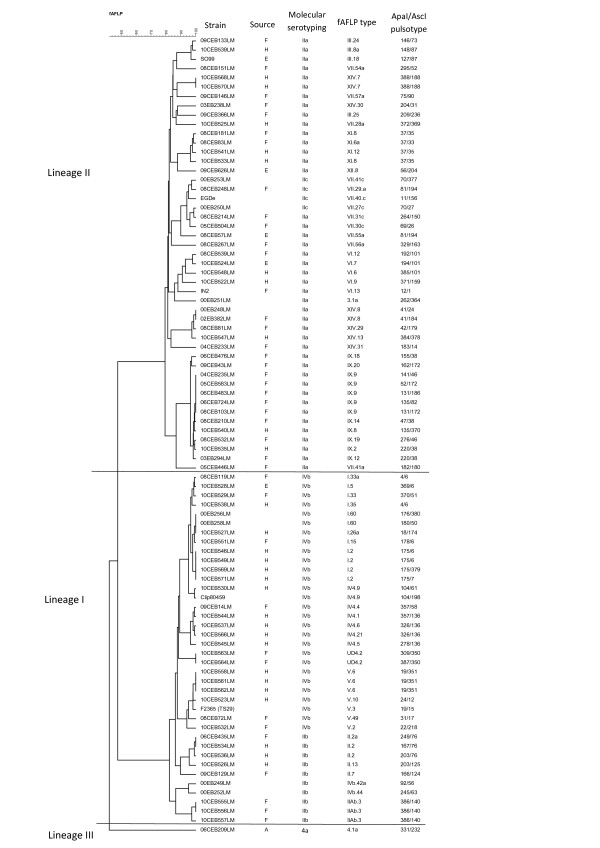
**Dendogram of similarity for 86 *L. monocytogenes *isolates based on fAFLP type using the Dice coefficient and UPGMA.**

Each fAFLP and PFGE type contained isolates belonging to only one of the 4 molecular serogroups, or serotype 4a, except for one PFGE type (81/194) which contained isolates from serogroups IIa and IIc (Figure 1).

### Reproducibility and discriminatory power of the subtyping methods

Table 1 shows the subtyping results of isolates used to evaluate the reproducibility, the discriminatory power and the ability to recognize same-type groups of isolates using PFGE and fAFLP. Isolates included in the study as duplicates gave indistinguishable fAFLP types and PFGE types (Table 1). Table 1 also shows that distinct PFGE types and fAFLP types were observed in each groups of isolates associated with outbreak or sporadic cases, except for TS isolates group 03: PFGE type 120/191 was detected in *L. monocytogenes* TS67, TS56 (duplicate of TS77) and TS 39, but displayed two different fAFLP types *i.e.* VII.27 and VII.27a. These 2 fAFLP types were indistinguishable except for a small additional ‘shoulder’ after a double peak of 206 base pairs, as seen on the PeakScanner scan, present in strains TS39 and TS67 (type VIIa.27a) but not in isolate TS56 (type VIIa.27). To rule out any fluorescent artefacts, the 3 isolates were processed in triplicate on separate occasions and the fAFLP profile obtained by each replicate was always the same, including the ‘shoulder’ at 206 bp with strains TS39 and TS67.

**Figure 2:**
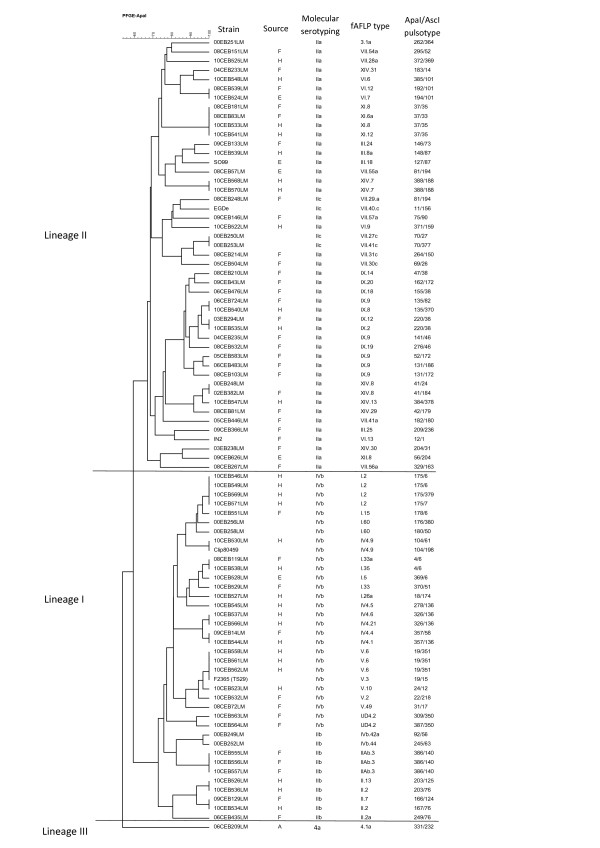
**Dendogram of similarity for 86 *L. monocytogenes *isolates based on *Apa*I-PFGE type using the Dice coefficient and UPGMA.**

**Figure 3:**
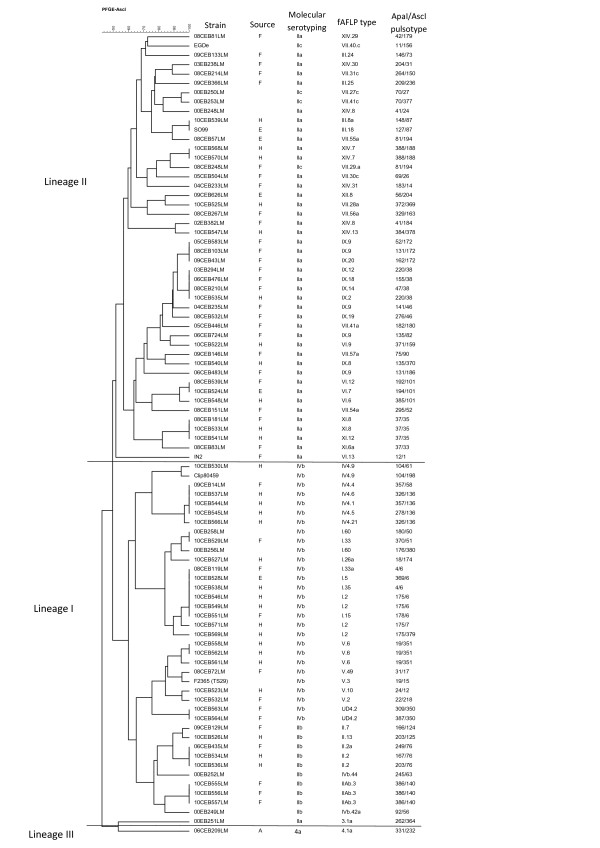
**Dendogram of similarity for 86 *L. monocytogenes *isolates based on *Asc*I-PFGE type using the Dice coefficient and UPGMA.** H: human, F: food ; E: environment ; A: animal.

Both subtyping methods separated the isolates into three distinct groups correlating with *L. monocytogenes* genetic lineages I, II and III (Figure 1; Figure 2; Figure 3). The 11 reference strains, including the 8 CLIP and the 3 fully sequenced strains, were classified by both fAFLP and PFGE, into the expected genetic lineages (Figure 1; Figure 2; Figure 3). The discriminatory power of fAFLP and PFGE was evaluated using 97 isolates including field strains, references strains, sporadic cases and representative isolates from each outbreak. The ID calculated from the typing results of fAFLP and PFGE is shown in Table 3. The ID calculated from fAFLP typing was 0.993 and from PFGE typing 0.996. Both typing techniques were found to be more discriminatory for *L. monocytogenes* Lineage II than for those of lineage I.

**Table 3:** **PFGE and fAFLP typing results from a panel of 97 *L. monocytogenes *isolates with index of discrimination (ID)**

***L. monocytogenes*lineages**	**Serogroups**^**1**^**or serotype**^**2**^	**Number of isolates**	**Number of PFGE**^**3**^**types**	**PFGE ID**^**4**^	**Number of fAFLP**^**3**^**types**	**fAFLP ID**^**4**^
I	IVb	35	36	0.988	33	0.981
	IIb	11				
II	IIa	45	45	0.995	43	0.989
	IIc	5				
III	4a	1	1	n/a	1	n/a
**Total:**		**97**	**82**	**0.996**	**76**	**0.993**

^1^ Serogrouping performed by multiplex PCR [4]: results are from both the European Reference Laboratory (EURL) for *L. monocytogenes* and the UK National Reference laboratory (UK-NRL) for *Listeria.*

^2^ Based on sero-agglutination performed by EURL.

^3^*Apa*I/*Asc*I-PFGE was performed by the EURL and *Hind*III/*Hha*I-fAFLP by UK-NRL.

^4^ Index of discrimination calculated according to the Simpson’s index of diversity (ID) [25]. n/a: not applicable.

## Discussion

The objective of this study was to compare fAFLP with PFGE for the subtyping of *L. monocytogenes*. The EURL for *L. monocytogenes* is the leader laboratory for improving or evaluate new typing methods and deploy them through the European NRL network. As well as comparing two subtyping methods, this study was also an opportunity to evaluate the inter-laboratory reproducibility of the multiplex PCR developed by Doumith et al. (2004) [4], to serogroup *L. monocytogenes.* The molecular serogrouping results of 109 isolates tested in this study were concordant between the two laboratories. The variant profile of serogroup IVb, characterized by the amplification of a supplementary gene fragment and previously described [26,27], was identified in the same four isolates by both laboratories, demonstrating the reproducibility of the method.

PFGE is widely acknowledged to be a time-consuming and labor-intensive method: the analyses are completed in 30 hours to three days from receipt of pure culture. It also requires highly skilled operators and does not offer commercially available standardized reagents. FAFLP has some advantages over PFGE: results can be achieved within 48 hours; the method is easy to perform and is less-labor intensive. It enables a high sample throughput and is readily automatable and standardization can be facilitated by the use of commercially available reagents. The cost per isolate for both techniques was calculated by the EURL and UK-NRL and was found similar: PFGE €6.02 and fAFLP £3.26. One inconvenience of fAFLP is the use of a capillary electrophoresis system such as a DNA sequencer to enable amplified fragments to be sized rapidly and accurately. However, the method could easily be used by laboratories currently performing PFGE, even those without a capillary electrophoresis equipment as many commercial companies now provide fragment analysis as a standalone service. As well as PFGE results, FAFLP data are suitable for electronic transmission between laboratories. FAFLP profiles could be prone to subjective interpretation in a similar manner to PFGE profiles with the generation of large, double and uncertain peaks. This was found to be the case when fAFLP was used for subtyping *Salmonella enterica*[28]. Therefore the choice of restriction enzymes is important. For *L. monocytogenes*, the fAFLP protocol used here was based on the digestion of bacterial genome by the restriction enzymes *Hind*III and *Hha*I. This combination of enzymes generated profiles typically composed of between 50–80 fragments within a range of 60–600 bp, which were easily recognisable as fluorescent peaks on PEAK SCANNER™ chromatographs. The level of fluorescence sometimes varied between different batches of samples, but the number of peaks obtained by replicate samples in different batches was 100% reproducible. Therefore, the percentage of similarity between each fAFLP types selected was higher (100%) than chosen in previous works (>95%) [11,13].

The 109 isolates were divided by fAFLP and PFGE into three clearly distinguishable lineages. A similar division had previously been detected by fAFLP analyses with enzyme combinations other than those used in this study [9,10]. This division correlates with the flagellar (H) antigen type which confirms the phylogenetic divergence between strains of serogroups IVb and IIb and those of serogroups IIa and IIc.

The subtyping results obtained in this study on a panel of *L. monocytogenes* field strains from human clinical cases, foods, food processing environments and animal cases, reference strains and isolates associated with outbreaks or sporadic cases showed equal discriminatory ability between fAFLP (ID 0.993) and *Apa*I/ *AscI*-PFGE (ID 0.996). Lomonaco et al. (2011) [13] also obtained similar discriminatory power between these 2 subtyping methods, but only on a panel of *L. monocytogenes* isolates from environmental and food sources. With other bacteria such as *Salmonella* and *E.coli* 0157, the discriminatory power of fAFLP was also found to be similar to PFGE [28].

In this study, isolates TS39 and TS67, produced a fAFLP profile indistinguishable from that produced by TS56 (duplicate of TS77), except for a small ‘shoulder’ after a specific double peak. The shoulder was not an artefact and appeared consistently, as shown by replicate testing. Because this difference was estimated as being ‘less than a peak’, all 4 isolates were assigned the same fAFLP type (VII.27) but for stringency purposes, the appendix ‘a’ was added to express the presence of the shoulder. These TS isolates were reported as a single type group (group 03) [17,20] according to the same Multilocus Enzyme Electrophoresis type by Pinner et al. (1992) [18]. However, in a separate study, PFGE profiles performed with adifferent combination of enzymes (*Apa*I/ *Sma*I) than those used by the EURL, showed the 2 isolates TS39 and TS67 to be closely related but different from TS56 [5]. Since PFGE and fAFLP rely on the recognition of restriction sites and therefore detect genetic variations on sections of the whole bacterial genome, whole genome sequencing would be a method of choice to reveal the difference between these isolates.

## Conclusions

In conclusion the UK-NRL fAFLP protocol has been shown to be highly discriminatory, equal to that of the EURL PFGE protocol. FAFLP can be used for investigating outbreaks of human listeriosis and tracking the source of contamination in foods and food processing facilities. This study demonstrated that the fAFLP protocol used by UK-NRL is an ideal alternative to PFGE to subtype *L. monocytogenes.* However, before deploying fAFLP through the European NRL network, this method needs to be fully standardized and its reproducibility assessed by proficiency test trials. This would enable efficient comparison and interpretation of fAFLP data and an agreed assignment of fAFLP types in the future.

## Competing interests

The authors declare that they have no financial end no-financial competing interests.

## Authors’ contributions

SR participated in the design and coordination of the study, the data interpretation and in drafting the manuscript. BF participated to the data interpretation step under BioNumerics software. KG conceived of the study and largely assisted in drafting the manuscript. TTD carried out all the PFGE and molecular serotyping tests at EURL. AB took part in drafting the manuscript. CA participated in the design and coordination of the study, carried out all the fAFLP and molecular serotyping tests at the UK NRL and helped draft the manuscript. All authors read and approved the final manuscript.
